# How Subtalar Kinematics Affects Knee Laxity in Soccer Players After Anterior Cruciate Ligament Injury?

**DOI:** 10.7759/cureus.47850

**Published:** 2023-10-28

**Authors:** Georgios Kakavas, Nikolaos G Malliaropoulos, Florian Forelli, Jean Mazeas, George Skarpas, Nicola Maffuli

**Affiliations:** 1 Sports Science and Physical Education, University of Thessaly, Trikala, GRC; 2 Sport Medicine, Fysiotek Spine and Sport, Athens, GRC; 3 Centre of Sports and Exercise Medicine, Queen Mary University of London Medical School, London, GBR; 4 Research and Development, Société Française des Masseurs Kinésithérapeutes du Sport (SFMKS) Lab, Pierrefitte sur Seine, FRA; 5 Orthopaedic Surgery, Clinic of Domont, Domont, FRA; 6 Sports Medicine, Orthosport Rehab Center, Domont, FRA; 7 Sports Injuries and Regenerative Medicine, “MITERA” General Hospital-HHG, Athens, GRC

**Keywords:** overpronation, graft laxity, subtalar joint, injury prevention, anterior cruciate ligament

## Abstract

Purpose

The goal of the current study was to ascertain whether there is an association between foot pronation and anterior cruciate ligament (ACL) injury in a group of elite professional soccer players.

Methods

Two groups of soccer players were studied, all of whom played in the Greek Super League. The ACL group included players who had suffered an ACL injury in the last 2 years. The non-ACL group was composed of players who had never suffered an ACL injury. We used a 3D baropodometric laser scanner to measure pronation or overpronation (navicular drop phenomenon) of the subtalar joint and how this affects the subtalar joint while standing. We assessed ACL laxity using the Genourob Rotab.

Results

ACL-injured patients, regardless of the mechanism of injury, exhibited greater navicular drop values than a randomly selected group of subjects with no history of ACL injury.

Conclusion

Greater knee joint laxity and subtalar pronation may be associated with an increased risk of ACL injury. Pronation of the foot appears to be a risk factor for ACL injury. These findings should be integrated into future studies to better define how neuromuscular control related to lower extremity biomechanics is associated with ACL injury.

## Introduction

Non-contact anterior cruciate ligament (ACL) injuries are common [1], with the highest incidence occurring in individuals aged 15 to 25 who participate in pivoting sports [2]. With an estimated cost of nearly a billion dollars per year [3], identifying risk factors and developing risk reduction strategies is both a scientific and public health priority [4].

The National Collegiate Athletic Association Injury Surveillance System recorded in 2004 that the highest ACL injury rate was found in American football for men [5,6]. Similar data was observed in gymnastics for women, with 33 injuries per athlete exposure. Current epidemiology indicates that ACL injuries constitute a larger proportion of total injuries for women than for men (3.1% vs. 1.9%), reaching as high as 4.9% of injuries in specific sports such as women's basketball and gymnastics [7]. The risk factors for non-contact ACL injuries fall into four distinct categories: anatomic, biomechanical, environmental, and hormonal [8]. This investigation aims to determine whether overpronation of the foot at the subtalar joint can affect ACL laxity and whether overpronation is associated with ACL injury.

## Materials and methods

All procedures described in the present study were carried out in accordance with the Declaration of Helsinki and were approved by the Institutional Review Board of the Physical Education Department of the University of Athens (IRB-N: 17654B).

**Figure 1:**
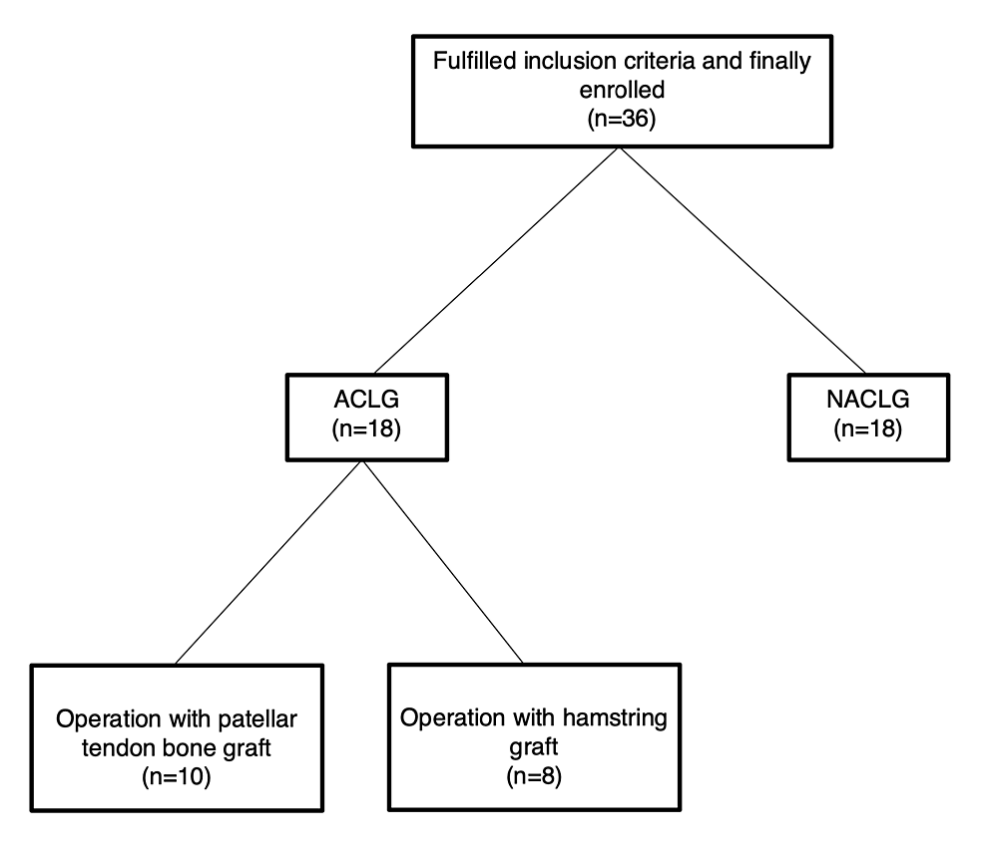
Strobe Flowchart ACLG: anterior cruciate ligament group; NACLG: non-anterior cruciate ligament group

All 36 soccer players who gave informed consent to participate in the present investigation were elite professional athletes aged 18 to 29 playing in the Greek Super League (Figure 1). These soccer players were divided into two groups of 18 soccer male players each, according to whether they had suffered an ACL injury in the last two years (ACL group), or had never suffered an ACL injury (non-ACL group). We used a 3D baropodometer laser scanner [9,10] to measure pronation of the foot (pronation index) (Foot Levelers Inc. 3D Body View Scanner, Virginia). We measured ACL laxity (ACL laximetry) with the Genourob Rotab (Genourob LLC, Paris, France) device. During their visit to the biomechanics laboratory, each subject underwent measurements, on both lower limbs. This included static assessment of both feet on a 3D body view scanner, with the eyes open, and weight bearing on both feet. It was measured and ensured that the body mass was equally distributed between the two feet. The 3D body view scanner has an accuracy of 300 microns. In each athlete, both feet were examined. The athlete stood still on the 3D scanner and the laser beam scans the feet one at a time. Additionally, comparative knee laxity was measured with the knee flexed to 30 degrees. Starting with one knee on a random basis, each knee underwent automated dynamic laximetry for anterior tibial translation and tibial rotation with 134N of force [11].

Inclusion criteria

The ACL group consisted of 18 male soccer players who suffered a magnetic resonance imaging (MRI) confirmed ACL tear in the last two years before the study, and had fully recovered following ACL reconstruction and were playing soccer. A patellar tendon bone graft had been used in 10 of them, and a hamstring graft had been used in the remaining eight players. The non-ACL group consisted of 18 male soccer players who had never suffered an ACL or other knee injury.

Exclusion criteria 

Players who had suffered an ACL injury less than two years for the ACL group, or for the non-ACL group, any injury to the knee and/or ankle over the last two years were excluded. 

Data analysis

Continuous variables were presented using summary statistics (number of patients with available observations (n_pt_), mean, standard deviation, median, 25th and 75th percentiles, minimum and maximum values). The distribution of continuous variables was also presented graphically using boxplots. Differences in the values of continuous variables between the ACL group and non-ACL group were analyzed using the Mann-Whitney U-test. 

## Results

**Figure 2:**
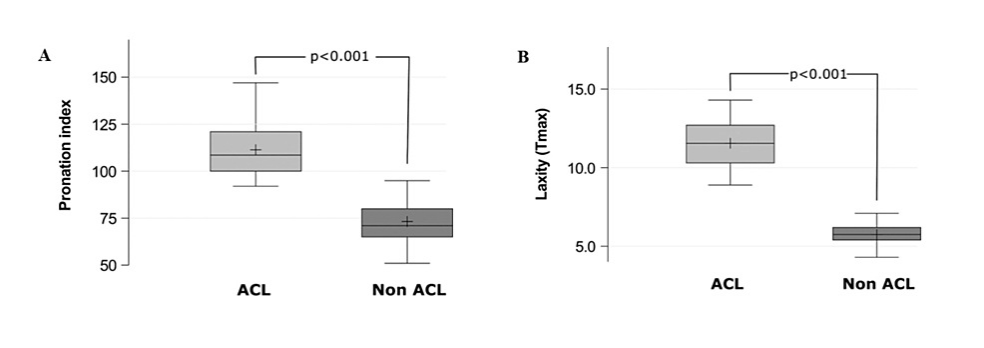
Differences in pronation index (Α) and laxity (Β) values between the ACL and non-ACL group. ACL: anterior cruciate ligament.

Table 1 presents the summary statistics for the pronation index and comparative laxity values for the ACL group and non-ACL group. Higher pronation index values were observed in the ACLG compared to the NACLG (median (IQR): 108.5 (100.0-121.0) vs 71.0 (65.0-80.0), p<0.001; Table 1, Figure 2). Similarly, higher laxity values (Tmax) were observed in the ACL group compared to the non-ACL group (Median (IQR): 11.6 (10.3-12.7) vs 5.8 (5.4-6.2), p<0.001; Table 1, Figure 2). 

**Table 1:** Differences in pronation index and laxity values between the ACL group and non ACL group ^a^Wilcoxon rank sum test. ACL: anterior cruciate ligament; SD: standard deviation; IQR:  interquartile range; n_pt_: number of patient.

		ACL group	Non-ACL group	p-value^a^
Pronation index	n_pt_	18	15	<0.001
	Mean (SD)	111.4 (14.7)	73.2 (12.1)	
	Median	108.5	71.0	
	Min-Max	92.0-147.0	51.0-95.0	
	IQR	100.0-121.0	65.0-80.0	
Laxity (Tmax)	n_pt_	18	15	<0.001
	Mean (SD)	11.6 (1.5)	5.7 (0.8)	
	Median	11.6	5.8	
	Min-Max	8.9-14.3	4.3-7.1	
	IQR	10.3-12.7	5.4-6.2	

## Discussion

The main finding of the present study is that elite professional soccer players who suffered an ACL injury had been operated and had successfully returned to sports exhibit significantly greater pronation of their feet when compared to a control group of elite professional soccer players who had never suffered such injury. Therefore, overpronation of the foot and internal rotation of the tibia may produce stresses on the ACL and predispose it to injury when rotational forces are imposed on the knee [5].

These results are in agreement with a prior report that ACL-injured patients, regardless of the mechanism of injury, exhibit greater navicular drop measures than subjects with no history of ACL injury [2]. Increased foot pronation is associated with increased medial tibial rotation [1,5]. Internal rotation of the tibia on the femur increases the strain on the ACL [12], increasing the risk of ACL injury [13], and is in line with observations on the increased incidence of non-contact ACL injury in athletes with pes planus [5,14-17]. In a study of navicular drop in a group of ACL-injured and uninjured subjects, there were significantly higher navicular drop test scores in the ACL-injured group [15]. Hyper-pronation and, therefore, the occurrence of ACL injuries may be related [18].

In a controlled study, Woodford-Rogers et al. showed that navicular drop, as well as anterior tibial translation, were higher in ACL-injured athletes compared to an uninjured group matched by sport, competitive level, and amount of time played [17].

However, it is uncertain whether hyper-pronation, as measured by navicular drop alone, is an adequate predictor of non-contact ACL injuries.

As the foot excessively pronates, eversion of the subtalar joint leads to obligatory internal rotation of the tibia. The femur naturally begins external rotation at the midstance phase of gait, when the tibia of the pronated foot continues to internally rotate. The opposing rotatory torques of femoral external rotation and tibial internal rotation may stress the knee joint and stretch the structures that limit knee rotation [19]. Increased pronation is associated with greater internal rotation in the transverse plane at the knee [1], which places additional strain on the ACL during deceleration activities and increases the risk of rupture. The authors are unaware of any intervention studies that have examined the role of foot orthotics as a prophylactic means of preventing ACL ruptures in athletes who hyper-pronate.

Although biomechanical information is still unclear about this matter, a higher joint laxity may be correlated with an increased ACL injury risk, as it increases mid-foot loading and thus affects lower limb kinematics. Increased muscular strength and neuromuscular control may compensate for the negative effects of joint laxity and be part of a wider panel of ACL dynamic loading [14,15]. This biomechanical relationship is found in prevention programs aiming to lower peak ground reaction forces and knee valgus in landing and cutting movements by increasing knee flexion and muscle loading capacities [20].

Athletes with increased generalized joint laxity demonstrate increased midfoot loading that may affect lower extremity biomechanics and potentially increase ACL injury risk. Although laxity may increase the risk of ACL injury, particularly in females, it is unclear whether joint laxity can be modified [14,15,20]. However, it may be possible to indirectly overcome the adverse effects of joint laxity with increased muscle strength and heightened neuromuscular control. The biomechanical relationships of ACL loading with lower extremity kinematics and kinetics target training programs to be focused on increased knee flexion angle and reduction of peak impact ground reaction force and knee valgus moments during landing [18].

One of the main issues in ACL injury management remains its clinical assessment and diagnosis. Although clinical examination is still found as the primary tool used by surgeons, with Lachman and pivot shift tests being the most sensitive and specific manual assessments found, such clinical examinations come with limitations, such as subjective training and experience [11].

It is therefore crucial to cross-check these clinical examinations with medical imagery and instrumented laxity measurements, along with the patient's full medical history. This combination allows for a better diagnosis and medical decision on the correct treatments to present. Clinical examination remains one of the most common and important diagnostic procedures for the ACL-deficient knee. The Lachman and pivot shift tests are considered the most sensitive and specific manual clinical tests, respectively. However, clinical examination has some limitations: it is dependent on a surgeon's training, experience, and technique [11]. Therefore, medical history, instrumented laxity testing, imaging, and arthroscopy also play an important role in the diagnosis of ACL ruptures. In addition, clinical examination, instrumented laxity testing, and MRI are required in combination to obtain broader diagnostic certainty.

Objective stress radiography may also assist clinicians in both diagnosing and managing an ACL injury by providing objective measures of sagittal and rotatory laxity of the knee. These methods may be used to identify complete ACL ruptures from partial tears, in which case the absence of laxity-related functional impairments might lead patients to be candidates for nonoperative treatment [20].

The present study offers an original contribution to the understanding of laxity and instability at the human knee joint. Measurements were taken in a non-invasive fashion, confirming that it is important to measure the anteroposterior and rotational laxity of the uninjured contralateral knee in assessing the laxity of the injured knee.

The present study evidenced a statistically significant association between overpronation, anterior knee joint laxity, and ACL injury. There are limitations of the present study. For example, given the retrospective nature of the investigation, one cannot infer a cause-effect relationship. Also, the number of subjects involved is relatively small. However, only elite professional athletes were recruited for the study. In such a setting, it would be difficult to recruit more subjects and to perform randomized studies to ascertain whether a given intervention would benefit in terms of the effects on the rate of occurrence of ACL injuries. 

Despite these limitations, the descriptive data and discriminant analyses indicate clinically relevant differences in inherent knee joint laxity and foot biomechanics between ACL-injured and non-ACL-injured athletes.

Measures of navicular drop (overpronation) and anterior knee joint laxity can be easily obtained. Pronation can be limited by selecting appropriate footwear or with orthotics. If joint laxity is of concern, a training program can be prescribed to strengthen the muscles surrounding the knee and increase neuromuscular control. Thus, these risk factors can be identified during routine physical examination and potentially mitigated through orthotics management and exercise. Also, appropriate neuromuscular control can be introduced in the training routine to reduce the relative risk of ACL injury.

## Conclusions

Reduction of the risk of non-contact ACL injuries should include the use of modern biomedical technology that includes state-of-the-art laxity and pronation measures. Future studies are needed to understand how neuromuscular control is related to laxity and pronation measures and the lower extremity biomechanics associated with ACL injury.
